# Role of phagocyte extracellular traps during *Mycobacterium tuberculosis* infections and tuberculosis disease processes

**DOI:** 10.3389/fmicb.2023.983299

**Published:** 2023-07-10

**Authors:** María García-Bengoa, Marita Meurer, Ralph Goethe, Mahavir Singh, Rajko Reljic, Maren von Köckritz-Blickwede

**Affiliations:** ^1^Institute for Biochemistry, University of Veterinary Medicine Hannover, Hannover, Germany; ^2^Research Center for Emerging Infections and Zoonoses, University of Veterinary Medicine Hannover, Hannover, Germany; ^3^LIONEX Diagnostics and Therapeutics GmbH, Braunschweig, Germany; ^4^Institute for Microbiology, University of Veterinary Medicine Hannover, Hannover, Germany; ^5^Institute for Infection and Immunity, St George’s University of London, London, United Kingdom

**Keywords:** *Mycobacterium tuberculosis*, *M.tb*, neutrophil extracellular traps (NETs), macrophage extracellular traps (METs), neutrophils, macrophages

## Abstract

*Mycobacterium tuberculosis* (*M.tb*) infections remain one of the most significant causes of mortality worldwide. The current situation shows an emergence of new antibiotic-resistant strains making it difficult to control the tuberculosis (TB) disease. A large part of its success as a pathogen is due to its ability to persist for years or even decades without causing evident clinical manifestations. *M.tb* is highly successful in evading the host-defense by manipulating host-signalling pathways. Although macrophages are generally viewed as the key cell type involved in harboring *M.tb*, growing evidence shows that neutrophils also play a fundamental role. Both cells are known to act in multiple ways when encountering an invading pathogen, including phagocytosis, release of cytokines and chemokines, and oxidative burst. In addition, the formation of neutrophil extracellular traps (NETs) and macrophage extracellular traps (METs) has been described to contribute to *M.tb* infections. NETs/METs are extracellular DNA fibers with associated granule components, which are released upon activation of the cells by the pathogen or by pro-inflammatory mediators. On one hand, they can lead to a protective immune response by entrapment and killing of pathogens. However, on the other hand, they can also play a severe pathological role by inducing tissue damage. Extracellular traps (ETs) produced in the pulmonary alveoli can expand easily and expose tissue-damaging factors with detrimental effects. Since host-directed therapies offer a complementary strategy in TB, the knowledge of NET/MET formation is important for understanding potential protective versus detrimental pathways during innate immune signaling. In this review, we summarize the progress made in understanding the role of NETs/METs in the pathogenesis of TB.

## 1. Introduction

Tuberculosis (TB) is one of the oldest known human diseases and still the 13th cause of death worldwide with approximately 1.6 million deaths in 2021 (World Health Organization [WHO], 2022). TB is a chronic infectious disease caused by the tubercle bacillus *Mycobacterium tuberculosis* (*M.tb*). *M.tb* has the ability to persist in infected people for years or even decades while concurrently manifesting an absence of discernible clinical symptoms. This is one of the reasons why it is so successful in causing the disease and difficult to control.

A critical aspect of *M.tb*’s success as a pathogen seems to rely on its co-evolution and adaptation to the human immune system (Comas et al., 2010). Indeed, innate immune cells play a key role during *M.tb* infection. Among those, neutrophils seem to have an important immunomodulatory role besides their direct microbicidal effects (Tan et al., 2006; Steinwede et al., 2012; Filio-Rodríguez et al., 2017; Nancy Hilda and Das, 2018). Interestingly, neutrophils have been shown to participate in the control of infection by several mechanisms including phagocytosis, degranulation, reactive oxygen species (ROS) formation, and the release of neutrophil extracellular traps (NETs). The initial description of NETs was made by Brinkmann et al. (2004). They showed that activated neutrophils release nuclear DNA as a form of an extracellular web with associated histones and granule proteases which entrap and kill invading pathogens (Brinkmann et al., 2004). Further studies showed that an excessive NET release, however, can lead to detrimental effects. Hence, NET release can contribute to thrombosis, tissue damage, autoimmune diseases, and cancer, a phenomenon with high clinical relevance, as reviewed by Neumann et al. (2020). More recently, the discharge of extracellular traps (ETs) by macrophages has been reported to be involved in disease control (Wong and Jacobs, 2013). However, the exact role of the formation of ETs by neutrophils or macrophages in the host response during TB is still not fully understood.

This review aims to summarize the current knowledge of the role of ETs from neutrophils and macrophages during *M.tb* infection and highlight the need for more intensive future research on this topic.

## 2. TB: Key steps during infection

TB is today the second leading killer of adults due to a single infectious agent, only after COVID-19 (World Health Organization [WHO], 2022). TB disease is normally initiated by the deposition of *M.tb* contained in aerosol droplets onto lung alveolar surfaces where the mycobacterium is engulfed by resident alveolar macrophages. However, *M.tb* has evolved mechanisms to survive within the host. After *M.tb* infection, bacteria block macrophage-mediated killing by inhibiting phagosome-lysosome fusion (Welin et al., 2011; Podinovskaia et al., 2013). Consequently, the mycobacterium survives and starts to proliferate within macrophages, thus establishing the infection. Furthermore, human necrotic macrophages provide a niche for *M.tb* replication before being released into the extracellular medium where it continues to grow and infect surrounding cells (Lerner et al., 2017). Importantly, necrotic macrophages release signals that recruit neutrophils to the site of infection, as shown in *M.tb* Erdman-infected mice (Repasy et al., 2015), which initiates the establishment of granuloma after approximately 3 weeks following infection (Seiler et al., 2003).

After primary infection, some individuals overcome the disease or progress to latent tuberculosis infection (LTBI) without any symptoms, but having a 5–15% lifetime risk of developing active TB (ATB) sometime in their lives, with the highest risk 2 years after infection (Trauer et al., 2016). The eventual evolution of LTBI to ATB depends on the complex interplay between bacterial and host factors.

The typical granuloma in LTBI is a physical barrier of immune cells that contains the infection. Human granulomas are often structured in microenvironments that balance pro-inflammatory responses, more prominent in the center of granuloma with higher content of antimicrobial peptides and ROS, while the tissue surrounding the caseous center is marked by an anti-inflammatory environment (Marakalala et al., 2016). These organized structures attempt to diminish the infection by decreasing the availability of oxygen and nutrients, and accumulating immune effectors and low pH to eradicate the pathogen inside the granuloma. However, under some circumstance, *M.tb* may adapt their metabolism to the hypoxic state and persist over years in the granuloma. In fact, a substantial heterogeneity of granulomas in different stages occur in infected individuals with some granulomas developing sterilizing immunity and others experiencing uncontrolled bacterial growth, necrosis and inflammation. In this phase, neutrophils are crucial for the development of lung lesions during TB disease (Kisich et al., 2002; Cardona and Prats, 2016). However, there is still a lack of broad scientific consensus on whether neutrophils contribute to protective host responses or increase pathologic consequences.

## 3. Neutrophil recognition and activation of antimicrobial activity in TB

Neutrophils are the most abundant cell type in bronchio-alveolar lavage fluid and sputum from active TB patients (Eum et al., 2010). In addition, increased neutrophil counts as well as elevated plasma levels of nucleosomes and elastase, are present in patients with pulmonary TB compared to healthy controls (van der Meer et al., 2017). Similarly, other studies described neutrophilia to be associated with lung tissue damage in pulmonary TB (de Melo et al., 2019). In humans, neutrophils are quickly attracted to sites of mycobacterial infection (Corleis et al., 2012), probably by an IL-8 gradient (also known as CXCL-8) (Corleis et al., 2012; Hult et al., 2021). Migration of neutrophils can also be mediated by different cytokines, mycobacterial antigens, and intracellular components, which may be released into the extracellular space upon necrosis of *M.tb*-infected macrophages. Additionally, neutrophils were found to accumulate as early as 2 h post-infection at the site of injection in a guinea pig model (Filio-Rodríguez et al., 2017).

During infection, neutrophils can act on *M.tb* using their classical defense mechanisms like degranulation and phagocytosis. Phagocytosis by neutrophils has been shown to be a key mechanism of the effective innate immune response against *M.tb*. Indeed, Kisich et al. (2002) showed that human neutrophils from healthy individuals were able to kill *M.tb* within 1 h of phagocytosis independently of oxidative mechanisms. Furthermore, this effect was boosted in the presence of tumor necrosis factor alpha (TNF-α) (Kisich et al., 2002). Similarly, a genetically engineered zebrafish model showed that some activated neutrophils within the granuloma had the potential to rapidly kill *Mycobacterium marinum* (Yang et al., 2012). However, this phenomenon was dependent on ROS produced by NADPH-oxidase (Yang et al., 2012). In addition to oxidative mechanisms, oxidative burst by neutrophils leads to the release of neutrophil-derived granules and granule-associated bactericidal proteins that can act as chemoattractants for T-cells and immature dendritic cells (iDCs) (Blomgran and Ernst, 2011). Similarly, neutrophils that undergo apoptosis can be a source of antimicrobial agents for macrophages, altering their cytokine profile, hence contributing to a more efficient killing of intracellular *M.tb* (Tan et al., 2006). Of great importance, both neutrophil elastase (NE) and serine protease cathepsin G contribute to resistance against mycobacterial infection in mice during early phases of disease (Steinwede et al., 2012). In addition, serine protease cathepsin G has been shown to have an important antimicrobial activity within hypoxic regions of lung granulomas in mice, and reduce mycobacterial burden (Reece et al., 2010). Nevertheless, proteolytic enzymes released by degranulation of neutrophils may also cause the destruction of neighboring cells and the dissolution of tissue.

Neutrophil inflammatory immune responses are involved in defense against *M.tb*. They are mediated by Toll-like receptors (TLRs), Fc receptors, G-protein-coupled receptors, adhesion receptors and cytokine receptors such as those for TNF-α, IFN-γ, and IL-18, as well as by other inflammatory signalling molecules and bacterial products (Futosi et al., 2013; Lyadova, 2017). When activated, neutrophils are an important source of specific cytokines and can prompt the innate immune response by stimulation of other immune cell types, like macrophages and DCs. For instance, TNF-α from neutrophils contributes to killing of the mycobacterium through several mechanisms. Those include the formation of granuloma and the stimulation of neutrophil migration, degranulation, oxidative burst and secretory activity (Kisich et al., 2002). Furthermore, the chemokine IL-8 plays a key role in oxidative burst of neutrophils themselves. Neutrophils are known to secrete IL-1β, IL-1α, CXCL1/3/4/8 attracting monocyte factors, GM-CSF and MMP-8, that participate in the migration of other immune cells (Scapini et al., 2000; Sawant and McMurray, 2007). The lymphocyte receptor for programmed death (PD-1) interacts with its ligands, the programmed cell death ligands PD-L1 and PD-L2. In a study by Tezera et al. (2020) the blockage of PD-L1 by an antagonistic antibody to PD-L1 resulted in improved survival during murine sepsis (Zhang et al., 2010). PD-1 is expressed in *M.tb*-infected lung tissue (Tezera et al., 2020). Interestingly, neutrophils that express PD-L1 are present in higher proportion in patients with ATB to those with LTBI (McNab et al., 2011), suggesting a role for PD-L1 expression in limiting immune control (McNab et al., 2011). In line with this, levels of expression of PD-L1 were significantly decreased in neutrophils after BCG immunization of healthy humans, while different immune activation markers from neutrophils, such as CD11b and CD66b were up-regulated, suggesting that BCG vaccination promotes immune control (Moorlag et al., 2020). In agreement, enhanced levels of myeloperoxidase (MPO) were detected in neutrophils upon exposure to *M.tb* three months after BCG vaccination, indicating increased degranulation, which is important for pathogen clearance (Moorlag et al., 2020).

Neutrophils may also interact with different immune cells through ectosomes, vesicles that are released from the cell membrane and that can selectively bind to macrophages regulating their antimicrobial activity (González-Cano et al., 2010; Duarte et al., 2012). In this regard, human neutrophils were able to induce release of ectosomes as early as 10 minutes after *M.tb* infection (González-Cano et al., 2010).

**FIGURE 1:**
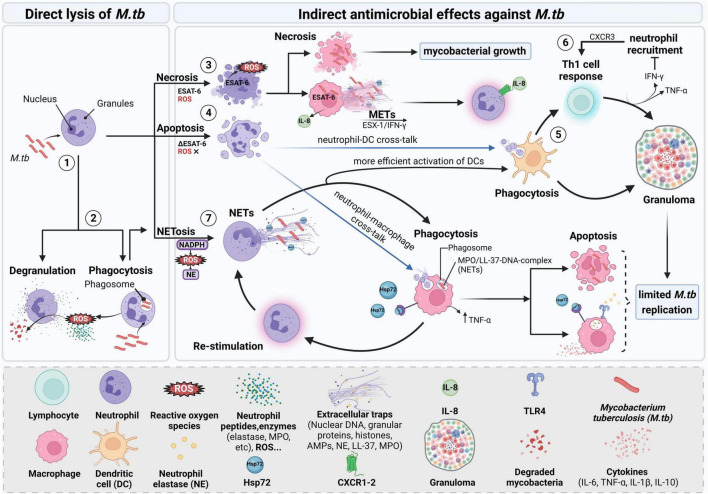
Mycobacterial uptake by neutrophils and interaction partners during infection. Neutrophils recognize and phagocytose the mycobacterium leading to successive events that include different immune cell types. (1, 2) After *M.tb* is engulfed, direct lysis includes NADPH oxidase starting to generate ROS which initiates granule degradation and subsequent release of NE. Neutrophils also release lysosomal enzymes and human neutrophil peptides lysing the mycobacterium. Bacteria that resist the direct oxidative killing will then be targeted by indirect antimicrobial effects that may involve the cooperation between different cell types. (3) Necrosis of neutrophils is dependent on ROS and ESAT-6. Removal of necrotic neutrophils by macrophages enhances *M.tb* replication and dissemination by inducing necrosis in macrophages. Activated macrophages can also form extracellular traps which can trap the microbe and prevent its further action on the host. METs are formed in an ESX-1-dependent manner mediated by levels of IFN-γ and involving the subsequent release of IL-8, thus activating neutrophils. (4) When inhibiting ROS and neutrophil necrosis, neutrophils may also undergo apoptosis restoring the ability of macrophages to control *M.tb* growth. (5) DCs can internalize pathogen antigens through apoptotic neutrophils cross-presenting them to T lymphocytes driving a protective Th1 response with subsequent release of IFN-γ and TNF-α, thus contributing to the maintenance of granuloma with IFN-γ avoiding excessive infiltration of neutrophils. (6) Additionally, neutrophils may participate by recruiting T cells via CXCR3-signaling chemokines. (7) For NETs to be formed upon *M.tb* infection, phagocytosis by neutrophils is essential. In the process of neutrophil activation, Hsp72 is released and NETs are formed. Elastase contained in NETs can activate macrophages and increase their capacity to kill the mycobacterium. Phagocytosis of apoptotic infected neutrophils and NETs by macrophages leads to the secretion of cytokines that signal other innate and adaptive immune cells for the elimination of the prevailing infection. Created with BioRender.com.

In summary, neutrophils are multitasking responders of the immune response to *M.tb* infections as summarized in Figure 1. Although neutrophils are known to play a key role in protection during the first 2 weeks of infection, as shown in experimental infections in mice (Tsai et al., 2006; Repasy et al., 2013), they seem to be cleared in later stages of TB, probably due to their short lifespan and contribution to inflammation (Tsai et al., 2006). Importantly, *M.tb* infected dying neutrophils need to be removed, since toxic molecules can be released to the surrounding tissues upon their death, leading to non-specific damage to host cells, known as necrotic lysis. The spent neutrophils are then cleared by efferocytosis, a process in which efferocytosing macrophages remove dying cells through specific surface receptors, contributing to host defense in TB (Andersson et al., 2020).

Different forms or phenotypes of neutrophil cell death are known: necrosis, apoptosis or NETosis. Alterations in neutrophil death may modulate neutrophil effector functions such as phagocytosis, cytokine release, or degranulation. A recent review briefly summarizes the various forms of neutrophil cell death including apoptosis, necrosis/necroptosis, and NETosis associated with the formation of so-called “neutrophil extracellular traps” (NETs) (Brostjan and Oehler, 2020). Importantly, factors mediating neutrophil cell death have been recently described to serve as potential biomarkers of pathological damage and disease progression, since an overwhelming immune response of neutrophils or massive neutrophil death can lead to detrimental consequences through released factors such as, for example, matrix-metalloproteinases (MMPs) (Ong et al., 2015).

A better understanding of the molecular basis of neutrophil death will help to identify targets for host-directed therapy (Fisher et al., 2022) to avoid detrimental consequences of neutrophil activation and/or cell death. Since the formation of neutrophil ETs is more recently discussed in the literature in the context of *M.tb* pathogenesis, our review focusses on the present knowledge of the role of NETs in the pathology of *M.tb* infection.

## 4. Neutrophils during granuloma formation in response to *M.tb* infection

*M.tb* is recognized for its adeptness in subverting and influencing host immune responses and cellular mechanisms to establish infection of the host, which also contributes to granuloma formation. Examples are the blockage of phagosomal maturation (Bach et al., 2008; Welin et al., 2008, 2011), cytosolic and membrane-bound compartments localization (Mwandumba et al., 2004), inhibition of autophagy (Romagnoli et al., 2012; Chen et al., 2015), manipulation of host cell death (Braian et al., 2013; Sharma et al., 2021), and neutralization of toxic components like ROS and toxic metals (Miller et al., 2010; Shin et al., 2010; Srivastava et al., 2019). Besides alveolar macrophages, neutrophils are regarded a predominant component of the innate immune response in TB (Desvignes and Ernst, 2009; Eum et al., 2010; Braian et al., 2013; Ong et al., 2015).

Most publications reviewing the formation of granuloma ignore the role of neutrophils, while focusing on macrophages as central components of this complex process. Nevertheless, neutrophils are known to be attracted by necrotic macrophages and to phagocytize the mycobacteria within these lesions, enhancing accumulation of these cells to the site of infection early during the formation of granuloma (Seiler et al., 2003). In this regard, TNF-α and IL-17 produced by neutrophils promote cell recruitment and granuloma organization throughout infection (Cavalcanti et al., 2012; Hu et al., 2017). Particularly, Hu et al. (2017) found that neutrophil-derived IL-17 inhibits *M.tb* growth through ROS production as well as neutrophil autonomous migration in the early phases of infection. In addition, neutrophils contributed to the establishment of granuloma in aerosol *M.tb* infected mice in an early stage, while restriction of the mycobacterial growth remained unchanged (Seiler et al., 2003). This was experimentally shown by the fact that the neutrophil population re-grows very fast after initial depletion in the first 4–5 days after aerosol infection with *M.tb* (Seiler et al., 2003). In this same study, the authors proved that neutrophils can regulate the granuloma via CXCR3-signalling chemokines, with increased levels of MIG (also known as CXCL9), a factor that attracts monocytes and T-cells, while no direct anti-microbial functions were detected. Since delayed development of granuloma did not affect mycobacterial growth and dissemination, it suggests that the process of granuloma formation may not be directly linked to preventing bacterial spread (Seiler et al., 2003). In a different study, neutrophils were described to establish close contacts with T-cells rather than contributing to clearance of infection in a so called “adaptive wave,” in which neutrophils were recruited to the site of infection for the second time (after the first wave) (Lombard et al., 2016). This was shown in a mouse model after intranasal infection with virulent *M.tb* (Lombard et al., 2016). Thus, neutrophils regulate the adaptive immune response during formation of granuloma. The central pro-inflammatory environment of granuloma is also linked to increased levels of neutrophils overexpressing granzyme B (grzB), as shown in human and macaque granulomas, a pro-apoptotic enzyme associated with cytotoxic T-cells (Mattila et al., 2015). Additionally, a correlation between higher neutrophil grzB expression and bacterial load was observed (Mattila et al., 2015). Furthermore, neutrophils expressing S100A9 (a calcium-binding protein of the S100 family, related to neutrophil extravasation and macrophage activation functions) have been associated with granuloma formation, as shown by abundant levels of S100A9^+^ neutrophils in the central area of granulomas in a guinea pig model of pulmonary TB, and in humans (Yoshioka et al., 2016).

In most instances, human TB granulomas have been shown to be hypoxic in patients with ATB (Belton et al., 2016). A common feature to all TB susceptible animals that develop hypoxic necrotizing granulomas, is the abundance of neutrophils (Turner et al., 2003; Mattila et al., 2015). In this regard, Marzo et al. (2014) observed a massive neutrophilic infiltration in a C3HeB/FeJ mouse model, which mimics the liquefaction (destroyed alveolar cell walls) of caseous necrosis occurring during active disease in immunocompetent adults.

However, the exact role of neutrophils during granuloma formation is still not entirely clear. Braian et al. (2013) hypothesized that NETs may play a vital role in the partnership between neutrophils and macrophages during granuloma formation in TB. Since NET-derived components like neutrophil-derived citrullinated histone H3 (cit-H3) or MMP-8 are elevated in samples from individuals with pulmonary TB (Ong et al., 2015; de Melo et al., 2019), NETs play a complex and context-dependent role in the TB pathogenesis, and this activity may also contribute to granuloma formation.

## 5. *Mycobacterium tuberculosis* and NETs

### 5.1. NET-formation and components of NETs

NET formation was first described in 2004 as an extracellular mechanism of neutrophils to entrap and partially kill microbes by immobilizing the pathogen to prevent its dissemination, and allowing high concentrations of antimicrobial agents to accumulate (Brinkmann et al., 2004). The major backbone of NETs is nuclear DNA, together with associated histones and additional antimicrobial components. Formation of NETs can be induced by different pathogens upon infection, as confirmed by several *in vitro* and *in vivo* studies. Thus, NET formation can be activated by bacteria (Brinkmann et al., 2004), viruses (Souza et al., 2018), fungi (Urban et al., 2006), protozoans (Guimarães-Costa et al., 2009), microbial componentes (Neeli et al., 2009), platelets (Clark et al., 2007), antigens (Oehmcke et al., 2009) and antibodies (Kessenbrock et al., 2009), as reviewed by Brinkmann et al. (2004), Fuchs et al. (2007), and von Köckritz-Blickwede and Nizet (2009).

NETs contain a wide arsenal of resources including enzymes (lysozyme, proteases), antimicrobial peptides (BPI, defensins, cathelicidins), ion chelators (calgranulin), and histones (Brinkmann et al., 2004; Brinkmann and Zychlinsky, 2012) for mediating bactericidal activity. Interestingly, MPO is essential for the killing of *Staphylococcus aureus* in a process that depends on the production of HOCI in the presence of H_2_O_2_ (Parker et al., 2012).

As described previously, formation of NETs might be a defense mechanism against microbes that are too big to be ingested by innate immune cells. Interestingly, Branzk et al. (2014) described NETosis as a mechanism that depends on particle size, by showing that a *Candida albicans* mutant that is “locked” in the yeast form without progressing to hyphae morphology, failed to induce NETs. Similar results were obtained in the same study with *Escherichia coli* and *Klebsiella pneumoniae*. However, neutrophils were able to release NETs upon 4 h incubation with aggregates of *Mycobacterium bovis* BCG. These findings suggest that when organisms are small enough to be phagocytosed, they do not induce NETosis (Branzk et al., 2014).

Since most neutrophils die when they release NETs, the term NETosis is used to define the initially described cell death mechanisms behind this phenomenon (Fuchs et al., 2007). However, nowadays, there are two predominant distinct NET formation pathways described: (1) the NADPH oxidase 2 (Nox 2)-dependent pathway associated with suicidal NETosis and disruption of the plasma membrane after several hours of stimulation (Fuchs et al., 2007), and (2) an early vital NET vesicular formation which occurs within minutes (Pilsczek et al., 2010). During vital NET-formation, NETs are released via nuclear envelope blebbing and vesicular export, maintaining the plasma membrane intact and allowing neutrophils to stay alive to perform other immune functions (Pilsczek et al., 2010; Rochael et al., 2015).

The classical mechanism of late suicidal NETosis, is characterized by the translocation of MPO and NE to the nucleus, degradation of histones, decondensation of the nucleus, a pre-lytic rupture of the nuclear membrane, and the subsequent mixing of cytoplasmatic, nuclear, and granule components, that are released as fibers to the extracellular environment. Finally, released NET fibers are composed of nuclear DNA, citrullinated histones, neutrophil granule proteins like NE and MPO, cationic antimicrobial peptides such as the cathelicidin LL-37, and matrix-metalloproteinases like MMP-8 and -9.

NADPH-oxidase-dependent production of ROS has been shown to be required for NETosis. Fuchs et al. (2007) observed that chronic granulomatous disease (CGD) patients with mutations in the NADPH-oxidase complex are not capable of producing ROS and subsequently NETs. In agreement, the usage of an inhibitor of NADPH oxidase enzymes, diphenylene iodonium, completely suppressed the formation of NET structures (Fuchs et al., 2007). In this regard, phorbol myristate acetate (PMA) is the most efficient inducer of the NADPH-oxidase enzymatic complex and therefore, a potent activator of NET release, since it stimulates protein kinase C in a specific way. Also, NE appears to be essential for NET formation as NET structures were not found in a NE-deficient mouse model (Papayannopoulos et al., 2010). Similarly, patients lacking MPO were not able to produce NETs (Metzler et al., 2011).

NADPH-oxidase 2 dependent NETosis often initiates in neutrophils with protein kinase C activation, triggering the Raf-MEK-ERK pathway. This pathway subsequently induces phosphorylation and triggers the activation of a subunit of NADPH oxidase 2 (NOX2), leading to ROS production (Hakkim et al., 2011). However, individual cases as e.g., induced by amoebas, also show that NET-formation may involve Raf/MEK/ERK, but is independent of protein kinase C and ROS (Fonseca et al., 2018). Thus, multiple signalling events can lead to a similar phenotype and morphological characteristics of NETosis.

More recently, gasdermin D (GSDMD) from neutrophils has been implicated in NET formation, and NE is essential for GSDMD to be activated, with both participating in a “feed-forward loop” (Sollberger et al., 2018). GSDMD is a pore-forming protein that facilitates formation of pores first into the granule membranes, with subsequent release of NE. NE later translocates into the nucleus where histones are processed, and following pores formation by GSDMD in the plasma membrane, allow the release of NETs into the extracellular environment. The role of GSDMD is emphasized by studies with LDC7559, a compound based on the pyrazolo-oxazepine scaffold, found to inhibit the formation of ROS-dependent NETs by specifically binding GSDMD (Sollberger et al., 2018). Furthermore, Chen et al. (2018) confirmed the results from Sollberger et al. (2018) by showing that GSDMD-dependent cell death induced NET-formation. Although classical NETosis occurs independently of caspases (Fuchs et al., 2007; Remijsen et al., 2011), Chen et al. (2018) described the non-canonical inflammasome signalling including caspase-11 to induce GSDMD-dependent NETosis that acts independent of MPO, NE and PAD4, but with similar morphological features to those from the classical caspase-independent NETosis pathway. The caspase-11-induced NETosis is regulated by GSDMD, and mediates host protection *in vivo* via the bactericidal effect mediated by the release of NETs, as shown in a caspase 11/GSDMD deficient mouse model in which the bacterial burden in the spleens was enhanced compared to the wild-type control (Chen et al., 2018). GSDMD is found in NETs, therefore it is possible that it plays a role in pathogen killing by contributing to NET release but also by its antimicrobial properties (Sollberger et al., 2018).

It is interesting to note that although caspase-11–induced NETosis and classical NE-driven NETosis are resulting in morphological similar phenotypes, they both are initiated in response to different signalling mechanisms but, centering on GSDMD as the common denominator. Then, these data reinforce the view that multiple signal events initiate NETosis.

Besides suicidal NETosis, NET formation can also be induced via a cascade of signalling events that initiate the active release of DNA-containing vesicles which retain neutrophil integrity and viability. This process is often termed as “vital NET formation” or “vesicular NETosis,” and often occurs independent of NADPH-mediated ROS-formation, within less than an hour upon stimulation. The activation of this process involves complement-mediated pathogen opsonization or signaling by TLRs (Clark et al., 2007; Yipp et al., 2012). In particular, TLR-4-activated platelets boost the vital NET formation as a process to immobilize bacteria in septic blood (Clark et al., 2007). Additionally, the TLR-2 and complement receptor 3, are involved in vesicular NET-release upon neutrophil stimulation with Gram-positive bacteria (Pilsczek et al., 2010). Importantly, during this process the plasma membrane is not damaged, with the consequence that neutrophils are still able to maintain their competence to migrate and phagocytose microbial invaders (Yipp et al., 2012). However, at later stages, the nuclear membrane also undergoes a complete rupture, which is then further causing accumulation of chromatin fibers and NETs in the cytosol (Pilsczek et al., 2010). Thus, vesicular NET formation may also be, somehow, at a certain stage or condition, associated with suicidal NETosis, as recently reviewed by von Köckritz-Blickwede and Winstel (2022).

Independent of the phenotype of NET-formation, cit-H3 is described as a specific marker for NETs in both cases. Cit-H3 is a histone with arginine residues post-translationally changed into citrullines by deimination, contributing to a more relaxed chromatin structure (Wang et al., 2009). Hypercitrullination of histones is a central process for NETosis and also vesicular NET release, as catalyzed by the peptidylarginine deiminase 4 (PAD4) that mediates chromatin decondensation and subsequent NET formation. In a study by Hemmers et al. (2011) mice lacking PAD4 were not able to form NETs, with undetectable levels of hypercitrullination of the core histone H3. Conversely, Li et al. (2010) concluded that PAD4 plays a role mainly in antibacterial innate immunity mediated by NETs, rather than histone-mediated bacterial killing.

### 5.2. NETs during pulmonary TB disease

**FIGURE 2:**
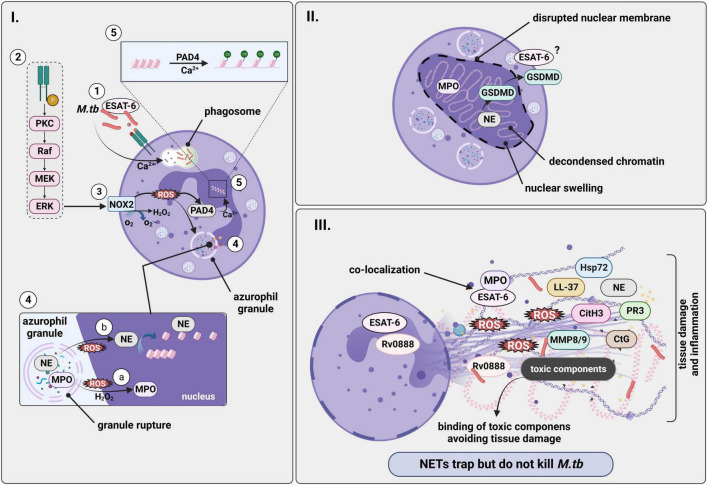
*M.tb* induces formation of NETs in a NADPH-oxidase dependent manner. Infection with *M.tb* promotes release of NETs. However, NETs trap but do not kill the mycobacterium. (I.1) NET formation begins with phagocytosis of *M.tb* by neutrophils leading to neutrophil activation in a Ca^2+^ dependent manner, with ESAT-6 being directly involved. (I.2) The process possibly continues with the activation of a cascade of intracellular enzymes such as protein kinase C and the Raf-MEK-ERK pathway, leading to the phosphorylation of the (I.3) NADPH-oxidase (NOX) which converts O_2_ to superoxide (O_2_^–^) and subsequent generation of ROS. (I.4) (a) A downstream product of NADPH oxidase activation, H_2_O_2_, acts as substrate for MPO, and ROS promotes translocation of both NE and MPO from granules to the nucleus where (b) NE mediates chromatin degradation and decondensation by binding to histones. (I.5) PAD4 would also contribute to decondensation of the chromatin by histone citrullination in a process that is dependent on Ca^2+^. (II) DNA extrusion and chromatin decondensation contributes to nuclear swelling and to the disruption of the nuclear membrane. Therefore, chromatin fills the cell and would be released as a NET upon cell lysis, a process in which pore-forming proteins such as GSDMD, which is activated by NE, also participate. (III) NETs are finally released into the extracellular space containing different granular proteins and histones associated to tissue damage, as well as bacteria and bacterial components such as ESAT-6 that would co-localize with MPO. On the other hand, NETs sequester the toxic contents from dying neutrophils preventing damage to surrounding tissue. Created with BioRender.com. *M.tb*, *Mycobacterium tuberculosis*; NADPH-oxidase, nicotinamide adenine dinucleotide phosphate oxidase; ROS, reactive oxygen species; NETs, neutrophil extracellular traps; ESAT-6, early secretory antigenic target 6; NE, neutrophil elastase; MPO, myeloperoxidase; PKC, protein kinase C; PAD4, peptidylarginine deiminase 4; GSDMD, gasdermin D.

Whereas Figure 1 highlights the general role of neutrophils and NETs in the pathology of *M.tb* infection, Figure 2 summarizes the molecular mechanisms behind *M.tb*-induced NET formation. The main literature is also indicated in the Supplementary Table.

*M.tb*-induced NETs depend on ROS generated by NADPH-oxidase, as well as NE (Braian et al., 2013; Su et al., 2019) and MPO (Schechter et al., 2017; Su et al., 2019). Similar to some other pathogens e.g., *C. albicans*, *M.tb*-induced NET formation relies on phagocytosis (Urban et al., 2006; Braian et al., 2013). Also for *M.tb* infection, NETs have antimicrobial effects and the potential to trap the bacteria, thus immobilizing the pathogen, which may lead to the prevention of mycobacterial spread. Importantly, thereby NETs may modulate the process of granuloma formation and immune control in TB since macrophages get activated after phagocytosis of NETs during the early multicellular inflammatory reaction (Braian et al., 2013). However, NETs are not able to kill *M.tb* either *in vivo* or *in vitro* (Ramos-Kichik et al., 2009; Corleis et al., 2012). Similar results were obtained by Ramos-Kichik et al. (2009) after incubation of *M. canettii* with human neutrophils *in vitro*. Interestingly, Repasy et al. (2013) reported NETs formed *in vivo* in *M.tb* infected lungs of both interferon-c knockout and hypercholesterolemic ApoE null mice, suggesting that there may be an extracellular population of *M.tb* bacteria in the lung that is trapped by NETs. It was also reported that the *M.tb*-NETs formation occurs in a time dependent manner, since longer incubation periods of bacteria and neutrophils lead to greater release of NETs (Ramos-Kichik et al., 2009). However, *M.tb*-induced NETs were shown to kill *Listeria monocytogenes*, another intracellular pathogen, confirming once more their antimicrobial effect, and indicating that *M.tb* is more resistant to NETs microbicidal activity (Ramos-Kichik et al., 2009).

Contrary to the theory proposing the protective effects of NETs, type I IFN-induced NETs are suggested to facilitate the extracellular growth of *M.tb* in TB-susceptible mice, thus contributing to pulmonary lesions and inflammation (Moreira-Teixeira et al., 2020). Furthermore, cit-H3, besides being a NET-marker as previously shown by others (Biron et al., 2017; Thålin et al., 2017; Kuczia et al., 2020), is also a potential marker for lung tissue damage (LTD) induced by severe pulmonary TB (de Melo et al., 2019). Thus, significantly higher levels of neutrophilia-related serum cit-H3 are present in LTD patients and correlate with increased cavity formation, resulting in high titers after 60 days of anti-TB treatment (de Melo et al., 2019). This suggests a central role for NET induction during TB. Overall, NETs might promote lung injury in neutrophilic TB lesions, as they were shown to do so during severe influenza infections (Narasaraju et al., 2011).

Importantly, MMP-8, one of the key factors involved in tissue damage during TB, is found to be present in high amounts in NETs after *M.tb* infection *in vitro*, and its levels are increased in sputum from TB patients compared to controls (Ong et al., 2015). Moreover, neutrophils containing elevated MMP-8 and MMP-9 are localized in the inner wall of cavities, in the central area of necrotic granulomas in patients with pulmonary TB, correlating with matrix destruction (Ong et al., 2015). Similarly, comparable levels of MMP-8/9 were observed when stimulating human neutrophils with conditioned media from monocytes infected with *M.tb* (CoMTB) (Ong et al., 2015). Further, Ong et al. (2018) investigated the effect of hypoxia on neutrophil-derived enzymes and tissue destruction in TB. Thus, hypoxia led to increased levels of neutrophil-derived MMP-8/9 when stimulating these cells with CoMTB (Ong et al., 2018). Additionally, gene expression levels of NE were also enhanced. However, *M.tb*-induced NET formation decreased by 32% during hypoxia compared to normoxia, in stimulated primary human neutrophils after 4 h stimulation (Ong et al., 2018). Furthermore, differences were not found when comparing neutrophil phagocytosis of *M.tb* during both normoxia or hypoxia, and hypoxia led to a delay in apoptosis of neutrophils and a reduced number of necrotic cells after infection with *M.tb* (Ong et al., 2018). However, the authors concluded that decreased levels of NETs during hypoxia were not due to a decrease in cell viability in neutrophils (Ong et al., 2018). Nevertheless, although MMP-8/9 expression is differentially regulated compared to formation of NETs, NETs might serve to bind and accumulate MMPs and thereby, trigger a local high concentration with subsequent detrimental tissue damage. Likewise, high MMP-8 levels are also found in patients with other respiratory diseases (Mattey et al., 2012).

The potential for detrimental effects of NETs during TB, highlights the need for their efficient elimination by the host to prevent further tissue damage. Macrophages can engulf spent neutrophils by efferocytosis and may be able to eliminate NETs, thus preventing tissue damage (Braian et al., 2013; Andersson et al., 2020). Phagocytosis of dying neutrophils by macrophages has been shown to decrease viability of intracellular *M.tb* (Dallenga et al., 2017). Besides, macrophages can acquire resulting antimicrobial molecules from dying neutrophils by phagocytosis of NETs, favoring the killing of *M.tb* (Braian et al., 2013). Recruited macrophages engulf mycobacteria and secrete a collection of proinflammatory cytokines and chemoattractants. Importantly, macrophages co-cultured with *M.tb* induced-NETs, secreted increased levels of IL-6, TNF-α, IL-1b, and IL-10 (Braian et al., 2013).

Additionally, it is important to mention that the heat shock protein 72 (Hsp72) plays an important role in the interaction between neutrophils and macrophages. Hsp72 was found to be highly abundant in NETs induced by *M.tb* (Braian et al., 2013). What is more, NETs binding Hsp72 or recombinant Hsp72, were able to trigger cytokine release of IL-6, TNF-α, IL-1β, and IL-10 from macrophages. However, when neutrophils were treated with the NADPH oxidase inhibitor diphenylene iodonium, NET formation was impaired and the *M.tb*-activated neutrophils lost their stimulatory properties. Nevertheless, if this effect is contributing to a protective defense in the host or causing detrimental effects, is still unclear.

In addition, human antimicrobial LL-37 is a pro-inflammatory cathelicidin belonging to a family of small cationic pore-forming peptides. It participates in the antimicrobial activity of NETs when it is highly and locally concentrated in these trapping-like structures (Chow et al., 2010; Neumann et al., 2014a). Furthermore, LL-37 can stabilize NETs and thereby, protect them against bacterial nuclease-mediated degradation (Neumann et al., 2014c). Importantly, NET-bound cathelicidin LL-37 as well as DNA:LL-37 complexes are internalized by human macrophages and transported to macrophage lysosomes, where DNA is degraded. Resultant LL-37 was shown to co-localize with intracellular mycobacteria (Stephan et al., 2016). In the same study, Stephan et al. (2016) reported that LL-37 from LL37:DNA complexes contributes to the mycobacterial clearance inside BCG infected human macrophages by integration into bacterial membranes, as demonstrated by electron microscopy. This supports the idea that phagocytosis of NETs by macrophages prevents NET-induced inflammation and autoimmunity, and indicates that a balanced communication between neutrophils, NETs and macrophages could be critical for a protective rather than detrimental outcome of NETs activity.

## 6. *M.tb* recognition by neutrophils

*M.tb* expresses a wide variety of virulence factors, which have been addressed in detail in several reviews (Forrellad et al., 2013; Sharma et al., 2017; Singh et al., 2017). Therefore, here we will focus on some selected relevant examples connected to the detection and recognition by neutrophils.

The cell wall structure of *M.tb* is unique among prokaryotes and is a major determinant of virulence for the bacterium and critical for its long-term persistence in the host’s environment and for the progression of disease (Alderwick et al., 2015). *M.tb* cell wall contains a high proportion of mycolic acids, from which trehalose-6,6-dimycolate and sulpholipids are specifically recognized by neutrophils (Lee et al., 2012; Miyake et al., 2013). Other lipids like phthiocerol dimycocerosates and phenolic glycolipids are the principal components of the outer membrane of *M.tb*. Phthiocerol dimycocerosates contribute to the cell wall permeability barrier, mediate receptor-dependent phagocytosis of *M.tb* and, as reported recently, contribute to host cell escape and necrosis (Forrellad et al., 2013). In addition, mannose-capped lipoarabinomannan plays a key role in virulence. Mannose-capped lipoarabinomannan was found to facilitate the entrance of *M.tb* inside neutrophils by binding to lactosylceramide (Lac-Cer)-enriched lipid rafts, therefore, disrupting signalling necessary for phagolysosome formation (Nakayama et al., 2016). Other immunomodulatory properties have been conferred to the lipoprotein LpqH (19-kDa lipoprotein antigen), which is involved in the maturation of DCs (Hertz et al., 2001). Furthermore, the exposure of neutrophils to the *M.tb* 19-kDa lipoprotein induces neutrophil priming and activation by TLR-2 recognition (Neufert et al., 2001).

Notably, *M.tb* has sophisticated bacterial secretion systems to transport proteins important for virulence and host immune responses. Type VII secretion (T7S) systems, also called ESX systems, are necessary to ensure infection. Two members of T7SS, ESX-1 and ESX-5, have been shown to be involved in virulence. ESX-1 mediates the secretion of important virulent factors that are absent in the BCG vaccine, such as the early secreted antigenic target protein-6 (ESAT-6) and the 10-kDa culture filtrate protein (CFP-10). ESAT-6 and CFP-10 genes are located in a segment called region of difference 1 (RD1) and depletion of RD1 in an *M.tb* mutant resulted in attenuation of virulence (Lewis et al., 2003). Importantly, ESAT-6 and CFP-10 have been described to participate in necrosis of *M.tb*-infected neutrophils (Hsu et al., 2003; Corleis et al., 2012; Welin et al., 2015).

### 6.1. *M.tb* factors mediating formation of NETs

Factors that mediate NET formation by *M.tb* are suggested to be associated to the RD1 locus encoding for ESX-1, since RD1 has been shown to be important for neutrophil interaction (Corleis et al., 2012). However, its precise role in NET formation is controversially discussed. The RD1 region prevents the killing of *M.tb* by inducing a premature cell death of the infected neutrophils in a ROS-dependent manner. Thus, an RD1-deletion mutant was found to fail to induce necrosis in neutrophils and was also killed by neutrophils compared to the H37Rv *M.tb* wild-type strain (Corleis et al., 2012). Therefore, *M.tb* virulence factors encoded within the RD1 region seem to be required for escaping from neutrophil killing. In this regard, Dallenga et al. (2017) showed in a different study that ESAT-6 is required for *M.tb* growth in neutrophils and macrophages, supporting survival in phagocytic cells, since a ΔESAT-6 *M.tb* mutant induced apoptosis of phagocytic macrophages contributing to growth control. Interestingly, necrosis by neutrophils depends on ROS and was blocked when inhibiting MPO, thus restoring the mechanisms for mycobacterial growth control by macrophages (Dallenga et al., 2017). Furthermore, Rojas-Espinosa et al. (2021) observed morphological and functional changes in the nuclei of neutrophils from blood from TB patients including DNA extrusion, chromatin decondensation and nuclear swelling, suggesting that *M.tb* infection may induce NETosis. Nevertheless, recombinant proteins ESAT-6 and CFP-10, while inducing nuclear changes in stimulated neutrophils, did not activate formation of NETs (Rojas-Espinosa et al., 2021). Conversely, Francis et al. (2014) suggested in their study that ESAT-6-treated necrotic neutrophils produced NETs in a Ca^2+^ dependent manner with co-localization of ESAT-6 and MPO, supporting a role for ESX-1 in NET formation. In addition, neutrophils responded to *M.tb* infection with the release of NETs as early as 30 minutes post-infection, being evident by 4 h post-inoculation in a guinea pig model (Filio-Rodríguez et al., 2017), although the role of ESX was not dissected in that report.

Table 1 summarizes the *M.tb* factors involved in the interaction with neutrophils and NETs.

**TABLE 1:** *M. tb* virulence factors affecting NETs/METs.

*M.tb* virulence factor	Effect	References
**ESAT-6**	- ESAT-6 causes necrosis of neutrophils. - ESAT-6 has a leukocidin-like action, promoting increased levels of intracellular Ca^2+^ in aging neutrophils leading to calpain activation. - ESAT-6-stimulated neutrophils produced NETs colocalizing with MPO in a Ca^2+^ dependent manner.	Francis et al., 2014
	- Essential for the formation of METs by human macrophages upon cord-forming *M.tb* infection.	Kalsum et al., 2017
	- ESAT-6 together with neutrophil-derived ROS is a prerequisite for *M.tb*-infected neutrophils to undergo necrosis. - When inhibiting necrosis of *M.tb* infected-neutrophils, mycobacterial growth was blocked. - ESAT-6 is essential for mycobacterial growth in macrophages and neutrophils. - ESAT-6-induced neutrophil necrosis drives early direct contact between *M.tb* and the phagosomal membrane within macrophages.	Dallenga et al., 2017
	- ESAT-6 induces nuclear changes in neutrophils such as DNA extrusion, chromatin decondensation and nuclear swelling, suggestive of NETosis. ESAT-6 nuclear changes in neutrophils were similar to those produced by sera from patients with ATB. - ESAT-6 resulted in a more potent inducer of nuclear changes in neutrophils than CFP-10, and were also comparable to those induced by PMA.	Rojas-Espinosa et al., 2021
	- An *M.tb* ESX-1 deletion mutant did not induce macrophage colony-stimulating factor (M-CSF)–differentiated macrophages to form ETs. - ESX-1 induces necrosis of macrophages and this is potentiated by human IFN-γ.	Wong and Jacobs, 2013
**CFP-10**	- Involved in the induction of nuclear changes in neutrophils (DNA extrusion, chromatin decondensation and nuclear swelling) similar to those induced by PMA, a positive control for NETosis, although to a lesser extent than ESAT-6.	Rojas-Espinosa et al., 2021
	- Induce release of intracellular Ca^2+^ in human neutrophils in a NADPH-oxidase dependent manner. - CFP-10 significantly induced migration of neutrophils and primed neutrophils triggered ROS production when stimulated with CFP-10.	Welin et al., 2015
**Rv0888 sphingomyelinase (SpmT)**	- MPO, citrullinated H3 and histone H4 were detected in the lung and bronchoalveolar lavage fluid of mice infected with recombinant Rv0888. - NETs-mediated lung injury promoted release of the inflammatory cytokines IL-6, TNF-α and IL-1β. - Rv0888 sphingomyelinase activity induced NETs in the lungs of mice in a ROS-dependent manner and *in vitro* in human neutrophils, contributing to lung injury. - Depletion of sphingomyelin by sphingomyelinase leads to an increase in the formation of NETs.	Dang et al., 2018
	- Enhances replication of *M.tb* in human macrophages.	Speer et al., 2015
	- Rv0888 is essential for *M.tb* to be phagocytosed.	Niekamp et al., 2021

Several pathogens have developed strategies to escape from NETs, such as secretion of DNases or inhibition of the cathelicidin activity. Interestingly, many Gram-positive bacteria secrete nucleases, thus degrading NETs and impairing neutrophil survival and resistance. Examples are *Streptococcus pyogenes* and *S. aureus* that avoid NET-mediated killing (Buchanan et al., 2006; Walker et al., 2007; Berends et al., 2010). Furthermore, virulent encapsulated *Streptococcus pneumoniae* also resists killing by NETs (Beiter et al., 2006). There is the speculation that *M.tb* also has the capacity to escape NETs, due to expressing the extracellular nuclease Rv0888 (Dang et al., 2016). Rv0888, a protein belonging to the endonuclease/exonuclease/phosphatase family, is involved in the lysis of erythrocytes, constituting the main hemolytic factor of *M.tb*. Due to its sphingomyelinase activity, this factor enables also the bacterium to use sphingomyelin as a source of essential nutrients like carbon, nitrogen and phosphorus for intracellular growth during infection (Speer et al., 2015; Niekamp et al., 2021). Interestingly, besides the predicted nuclease activity, Dang et al. (2018) managed to express recombinant *Mycobacterium smegmatis* Rv0888NS, as well as a D438A nuclease mutant, and a sphingomyelinase mutant H481N, and observed formation of NETs exclusively in the lung tissues of mice administered with either recombinant *M. smegmatis* Rv0888NS or D438A. In agreement with this, levels of ROS and ceramide were found to be higher in those mice compared to H481N mutant and control groups, as determined in serum and lung homogenates (Dang et al., 2018). In the same study, human neutrophils from healthy human donors were stimulated with the respective recombinant *M. smegmatis* proteins, and NET structures were observed only when neutrophils were incubated with Rv0888NS and D438A, but not with H481N. All together, these findings suggest that formation of NETs does not occur upon exposure of neutrophils to the nuclease activity of the Rv0888 heterologously expressed in *M. smegmatis* (Dang et al., 2016, 2018). In contrast, recombinant Rv0888 sphingomyelinase activity induced NETs *in vivo* in the lungs of C57BL/6 mice in a ROS-dependent manner, and *in vitro* in human blood derived neutrophils (Dang et al., 2018). Hence, Rv0888 seems to be rather another NET formation inducing factor of *M.tb*. This is supported by Neumann et al. (2014b) who showed that neutrophils treatment with sphingomyelinase led to enhanced NET release.

Overall, the ability and efficiency of *M.tb* to induce NETs might highly depend on its virulence or immune evasion factor expression. However, from most studies it is evident, that *M.tb* is quite resistent to NET-mediated killing.

## 7. *M.tb*, macrophages and METs

**FIGURE 3:**
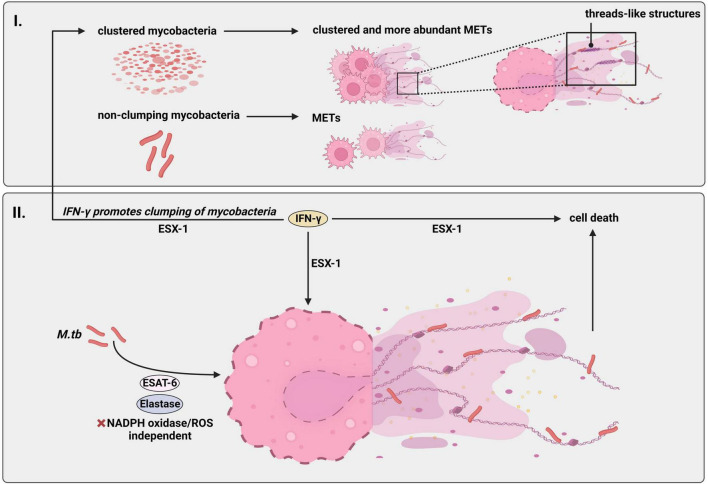
*M.tb* induces formation of METs independent of NADPH-oxidase and ROS. Human macrophages release METs upon infection with *M.tb*. (I) This effect is specially boosted in presence of the so called “cording” or clustered phenotype, that is when the mycobacterium accumulates and clumps together occupying a bigger volume compared to when the bacteria appear disseminated (“non-cording *M.tb*”). Notably, “cording-*M.tb*” induces formation of METs in form of threads. (II) Similarly to NETs, *M.tb*-induced METs are of nuclear origin containing double stranded DNA and citrullinated histones in their composition and *M.tb* can be found binding to DNA fibers. Furthermore, the mechanism of MET-formation following *M.tb* infection is regulated by antigen ESAT-6 since an *M.tb* ESAT-6 deletion mutant blocked METs. *M.tb*-METs are also regulated by enzyme elastase. However, this mechanism is independent of the enzyme complex NADPH-oxidase and ROS. Interestingly, IFN-γ enhances formation of *M.tb*-caused METs and induces aggregation of *M.tb* which in turn leads to increased formation of *M.tb*-METs, both dependent on ESX-1. Additionally, pre-treatment with IFN-γ promotes cell death of infected macrophages in a ESX-1-dependent manner. Thus, IFN-γ amplifies ESX-1 effects. Created with BioRender.com. *M.tb*, *Mycobacterium tuberculosis*; NADPH-oxidase, nicotinamide adenine dinucleotide phosphate oxidase; ROS, reactive oxygen species; METs, macrophage extracellular traps; ESAT-6, early secretory antigenic target 6; ESX-1, early secretory antigenic target 6 secretion system 1; IFN-γ, interferon-gamma.

Macrophages are well known as one of the first cell type arriving to the site of *M.tb* infection and for their ability to eliminate pathogens by phagocytosis. More recently, the formation and release of ETs by macrophages (METs) has been reported (Chow et al., 2010). METs formation was described to be regulated by molecular pathways similar to NETs (Chow et al., 2010). However, detailed investigations into mechanisms of MET release have been sparse. The knowledge on general MET-formation was recently reviewed by Doster et al. (2018) or Rasmussen and Hawkins (2022). METs can be indentified by the presence of typical ET markers such as e.g., MPO, MMPs or histones, among others (Chow et al., 2010; Wong and Jacobs, 2013; Halder et al., 2017; Kalsum et al., 2017). METs can also be formed in response to different stimuli as in the case of NETs, as reviewed by Doster et al. (2018). Interestingly, besides intracellular persistence in macrophages there is evidence that METs are also somehow induced by *M.tb* (Wong and Jacobs, 2013). Figure 3 summarizes the molecular mechanisms behind *M.tb*-induced MET formation. The main literature is also indicated in the Supplementary Table.

Wong and Jacobs reported for the first time that *M.tb*-induced macrophages participate in the release of ETs directly emerged from the nucleus and associated with cell death. They demonstrated that their structure was indeed similar to that in activated neutrophils (Wong and Jacobs, 2013). In the same study, they showed that METs induced by *M.tb* stimulation were regulated by elastase activities, and formed in a ESX-1 dependent manner since the formation of traps was blocked in an ΔESX-1 mutant (Wong and Jacobs, 2013). This was also shown in a different study by Kalsum et al. (2017) in which an ESAT-6 deletion mutant strain of *M.tb* did not form METs in human monocyte-derived macrophages. Furthermore, METs after *M.tb* infection were highly inducible by IFN-γ, a known mediator of antimycobacterial activity in murine macrophages, in an ESX-1 dependent manner (Wong and Jacobs, 2013). In this regard, IFN-γ pre-treatment also led to mycobacterial aggregation and necrotic death in macrophages infected with *M.tb*, whereas the same treatment had a minimal effect on macrophages infected with the *M.tb*-ΔESX-1 mutant (Wong and Jacobs, 2013). Interestingly, mycobacterial clumps more efficiently promoted ET formation than single bacteria, and macrophages with higher *M.tb* burden were prone to cluster together with extracellular traps during the progress of infection (Wong and Jacobs, 2013). Another study seems to point in the same direction. Thus, Kalsum et al. (2017) aimed to determine and characterize cord-forming *M.tb* induced-METs in human monocyte-derived macrophages, and were able to show that macrophages infected with non-cording bacteria produced relatively less METs compared to those released from cording-*M.tb*. Macrophages treated with the cording phenotype released ETs either in the form of threads or meshworks as visualized by electron microscopy. Together, both studies suggest that pathogen size may have an influence in the formation and release of NETs/METs. Although NETosis is known to be, in most cases, ROS-dependent as response to *M.tb* infection, the same is not true for METs, as diphenylene iodonium (a NADPH-oxidase-dependent ROS production inhibitor) did not show an inhibiting effect on MET production by human monocyte-derived macrophages (hMDMs) upon *M.tb* stimulation (Kalsum et al., 2017). These findings are in good correlation with other *in vitro* studies showing that MET formation is not dependent or linked to ROS and the NADPH-oxidase activity in response to cord-forming *M. abscessus* (Jönsson et al., 2013). ROS-independent formation of NETs is often associated with a vesicular NET-release (Pilsczek et al., 2010). However, such a morphological phenotype is until now not known for METs in response to *M.tb* infection.

Originally, ETs have been suggested as a protective mechanism that trap and possibly kill bacteria. However, the observation that ESAT-6 is required for the formation of METs suggests that it is beneficial for the bacteria (Kalsum et al., 2017). Finally, since MET-formation in response to *M.tb* infection is associated with cell death, this leads to unresolved inflammation, which is unable to reduce *M.tb* burden (Nathan and Ding, 2010). This highlights the need for a better understanding of the underlying mechanisms of MET-formation to develop possible treatment strategies that block this detrimental phenotype of macrophage activation.

## 8. Future perspectives of host-directed therapies including neutrophils, NETs and METs

The interactions between the host and *M.tb* are highly complex. Neutrophils have been shown to be phenotypically heterogeneous and to exert various functions during homeostasis and disease. Therefore, a better understanding of the neutrophil-pathogen interaction could shed light on disease mechanisms and help to develop potential new therapeutics. In addition, the different immune cells that are affected by neutrophils seem to differ based on TB disease stages and lesions. Apart from their direct effects upon infection with *M.tb*, neutrophils can mediate anti-inflammatory responses by releasing soluble mediators and NETs. With the increasing knowledge on NETs in TB pathogenesis, possible strategies or drugs that aim to modulate neutrophils or NETs could be useful for developing therapeutics for infection and disease control. In this context, host directed therapies (HDT) are potent TB therapies consisting of drugs that aim to suppress pro-inflammatory mechanisms to avoid excessive tissue damage and inflammation. Importantly, neutrophils are potent targets in HDT.

Neutrophils have been shown to play a detrimental role in promoting lung pathology during pulmonary TB, thus contributing to disease severity (Panteleev et al., 2017). Ibuprofen treatment, a non-corticoid anti-inflammatory drug, showed reduced levels of key enzymes involved in cellular processes for disease-control such as prostaglandin E2 synthesis and cyclooxygenase 2 in TB-susceptible mice, by strongly suppressing recruitment of neutrophils. Therefore, lesion size and mycobacterial loads were diminished when limiting neutrophil-mediated pathology in the lung throughout infection, confirming that neutrophils contribute to immunopathology of TB disease (Vilaplana et al., 2013). Similarly, Zileuton, a commercial 5-lipoxygenase inhibitor already approved for treating asthma, has been shown to reduce bacillary load and TB-induced lung damage in mice, while augmenting prostaglandin E2 (Mayer-Barber et al., 2014). Consequently, the inhibition of lipoxin biosynthesis may be a therapeutic approach to mycobacterial infection (Bafica et al., 2005). Furthermore, 1,25-dihydroxyvitamin D_3_ [1,25(OH)_2_D_3_], the active metabolite of Vitamin D, has diverse immunomodulatory properties for the control of *M.tb*. Vitamin D has been shown to enhance the expression of the antimicrobial peptide LL-37, the active form of human cathelicidin, which is expressed by various cells including neutrophils and macrophages, and thus, contributing to subsequent killing of intracellular *M.tb* (Coussens et al., 2009; Rekha et al., 2015, 2018). In addition, Vitamin D was shown to induce the production of ROS and nitrogen intermediates, while inhibiting *M.tb*-induced expression of metalloproteinases MMP-7, MMP-9, and MMP-10 in monocytes, facilitating the resolution of inflammatory responses (Liu et al., 2007). Hence, vitamin D supplementation could show beneficial effects in TB therapy by suppressing the pro-inflammatory response, and reducing the excessive tissue damage during active TB (Coussens et al., 2009). Importantly, the calcium-binding proteins S100A8/A9 (MRP8 and MRP14, respectively) expressed in neutrophils and monocytes, are known to accumulate in active TB granulomas from mice and macaques, and their expression seem to directly correlate with lung pathology (Gopal et al., 2013; Gideon et al., 2019). Therefore, they could be targeted as additional HDT for TB. In this context, previous studies by others proved mobilization of intracellular and extracellular calcium pools to be necessary to efficiently trigger NETosis when cells were incubated with IL-8 as physiological stimulus, since this inflammatory cytokine is known to contribute to calcium flux in neutrophils (Gupta et al., 2014). These results were further confirmed when calcineurin, a calcium-dependent serine/threonine protein phosphatase that is present in human neutrophils, was inactivated with the antagonist cyclosporine A (CsA) in IL-8 stimulated cells, resulting in the inhibition of the transport of calcium and, interestingly, a reduction in the formation of NETs (Gupta et al., 2014). Overall, avoiding excessive ROS generation, or NETosis by modulation of calcium transport, may help attenuate the excessive inflammatory response associated with TB. In furtherance of this knowledge, the presence of CsA impaired biofilm formation in *M. smegmatis* expressing PpiB, which is essential for this activity, suggesting that CsA physically interacts with PpiB (Kumar et al., 2019). Additionally, the authors showed that CsA also inhibits biofilm formation in *M.tb* through the same mechanism. *M.tb* biofilms can act as protective structures, making the bacteria less susceptible to the effects of antimicrobial agents. By inhibiting biofilm formation, CsA could enhance the accessibility of anti-TB drugs to the bacterium, increasing their effectiveness and potentially reducing the development of drug resistance (Kumar et al., 2019). Therefore, the potential use of CsA as an adjunct therapy to existing anti-tubercular drugs holds promise, as it targets multiple aspects of TB pathogenesis.

NET structures or NETosis are attractive targets for the development of therapeutics since their detrimental effects seem to predominate during active TB, especially when not efficiently eliminated by macrophages. In this regard, DNases are very effective at degrading NETs, however, several pro-inflammatory components such as histones and proteases associated to NETs, are also released upon DNA degradation leading to host tissue damage (Beiter et al., 2006; Buchanan et al., 2006; Kolaczkowska et al., 2015). Interestingly, anthracyclines, anti-neoplastic compounds that intercalate between base pairs and inhibit DNA replication and RNA transcription, have been shown to efficiently block NETosis through both the NOX-dependent and -independent pathways, without affecting bactericidal ROS production (Khan et al., 2019). Given the clinical relevance of NETs in TB pathogenesis, the more recently described LDC7559 (Sollberger et al., 2018), a potent inhibitor of ROS-dependent NET structures, may also represent a promising avenue for treating excessive release of NETs. LDC7559 binds to pore forming protein GSDMD and inhibits NET formation already at low concentrations, without interfering with phagocytosis, normal ROS production, and NE or MPO activity from neutrophils (Sollberger et al., 2018). Therefore, LDC7559 has potential as therapeutic agent directed to modulate detrimental NETosis while keeping neutrophil host defense mechanisms intact. Finally, therapeutic strategies directed to the inhibition of the PAD4 could block formation of NETs by avoiding citrullination of histones in both the NOX-dependent and -independent pathways (Hemmers et al., 2011). This is justified by a study by Kolaczkowska et al. (2015) in which PAD4-deficient mice showed 80% decreased NET release and significantly reduced tissue damage after methicillin-resistant *S. aureus* infection.

Since antimicrobial and immunomodulating peptides are well known to contribute to formation of ETs by neutrophils (Neumann et al., 2014a), it can be speculated that they might be promising targets for therapeutic interventions targeting NETs and/or METs. Hepcidin is a small peptide hormone secreted mainly by hepatocytes and highly expressed in response to infection or inflammation in TB (Olakanmi et al., 2007). This hormone turned out to not only regulate iron, but also has homology with peptides required for innate immune responses. Given the role of hepcidin in facilitating the recruitment of macrophages, it may be hypothesized that the peptide may have as well an effect on MET induction (Conceição-Silva et al., 2021). In a study by Zhang et al. (2021), it was shown that the formation of METs in adipose tissue was inhibited after blocking the expression of the hepcidin gene in a mouse model of diabetes. Importantly, hepcidin works as an antimicrobial peptide and has been recently shown to reduce the production of IFN-γ from T-cells, suggesting that modulation of hepcidin may be therapeutically useful for the control of TB (Tashiro et al., 2019).

## 9. Conclusion

Activated neutrophils use different mechanisms to fight *M.tb* infection. Though they do not have the capacity to efficiently kill the pathogen, neutrophils do have a huge impact in mediating immune responses by their interaction networks with other immune cells such as macrophages, and T cells, with different outcomes, from early clearance of infection to dissemination of viable bacteria. Importantly, NETs-formation is initiated as host’s early immune response to *M.tb* infection, which results in entrapment of the pathogen. NETs can trigger a proinflammatory cytokine response in adjacent macrophages during the early inflammatory reaction and thereby potentially also granuloma formation (Braian et al., 2013). However, the role of NETs in granuloma formation still remains to be elucidated. Apart from all antimicrobial repertoires of neutrophils and macrophages to eliminate the mycobacteria inside these cells, the release of ETs is an efficient mechanism to extracellularly fight these pathogens. Unfortunately, despite having these protective capacities, NET/METs can also lead to detrimental effects to the host, and therefore inhibiting extracellular traps may be beneficial to avoid tissue damage (de Buhr and von Köckritz-Blickwede, 2021). Accumulative evidence summarized in this review points toward massive detrimental effects of NETs and METs during *M.tb* pathogenesis. As a consequence, the use of nucleases may also be considered to possibly prevent excessive NET/METs formation during *M.tb* infection, as it was recently suggested for other severe lung infections such as COVID-19 disease (de Buhr and von Köckritz-Blickwede, 2021). Additionally, targeting NET/METs might be a promising future perspective for HDT to prevent long-lasting *M.tb* infection induced tissue damage and loss of function. The use of molecules or agents that target NET/METs can help in regulating the antimicrobial mechanisms of neutrophils and macrophages but without excessive inflammatory responses. Hepcidin might be another example to be tested in this context, since macrophages have been shown to over-express this peptide hormone in patients with severe pulmonary TB. However, whether hepcidin may attenuate the TB inflammatory response by also modulating METs and NETs is not known yet (Conceição-Silva et al., 2021) and should be investigated in future studies. Finally, understanding the role of NET/METs in the pathogenesis of TB may be crucial for identifying new treatment strategies for this very complex disease.

## Author contributions

MG-B drafted the first version. MG-B, MK-B, and MM performed literature search and analyzed the data. RG, RR, and MS provided revisions, comments, and additional literature review. MK-B, MM, and MS were responsible for supervision. All authors substantially contributed to the article, proofread the manuscript and approved the submitted version.
