# Supplementary material for: Role of phagocyte extracellular traps during Mycobacterium tuberculosis infections and tuberculosis disease processes
Source: Front Microbiol. 2023 Jul 10;14:983299. doi: 10.3389/fmicb.2023.983299 (PMC10365110; doi:10.3389/fmicb.2023.983299)
Supplement: Supplementary file 1 [file Data_Sheet_1.PDF]

Supplementary Table. ***In vitro* and *in vivo* studies on NET/METs in different stages of TB disease**

Effects/findings	ET type	Stimulus	*In vivo/ in vivo*	Associated mechanisms	Disease stage	Reference
• Protective effect of NETs in trapping *M.tb*. However, NETs correlates with disease severity suggesting a detrimental effect as well.<br>• *M.tb*-activated neutrophils acquired highly clustered NETs, more than those formed upon PMA stimulation.<br>• *M.tb*-induced NET formation is regulated by the NADPH-oxidase as well as NE.<br>• Upon *M.tb* stimulation, NETs bind and concentrate Hsp72 to the NET strands. Hsp72 is later used for the activation of macrophages triggering local cytokine production: IL-6, TNF-α, IL-1β and IL-10.	NET	*M.tb*	*In vitro* (human neutrophils andmonocytes isolated from healthy blood donors)	• Phagocytosis<br>• ROS	Healthy donors	(Braian et al., 2013)
• NETs are present in MPO positive neutrophilic lung lesions from infected C3HeB/FeJ mice as well as in necrotic lung lesions from patients withATB.<br>• Neutrophils isolated from patients with active TB over-express type I IFN-inducible genes.<br>• Increased type I IFN sig- nalling induces pulmonary NETosis and promotes mycobacterial growth in the absence of GM-CSF.	NET	*M.tb*	*In vivo* (infected C3HeB/FeJ TB-susceptible mice, and ATBpatients)	-	ATB	(Moreira-Teixeira et al., 2020)
• Patients with ATB show an aberrantly high level of low density granules that could be associated to increased NET release together with increased levels of ROS.	NET	*M.tb*	*In vitro* (LDGsand NDGs from ATB peripheral venous blood)	• ROS	ATB	(Su et al., 2019)
• ATB patients showed high levels of NETs and NE that correlates with plasma levels of nucleosomes.	NET	-	*In vivo* (plasma from ATB patients)	-	ATB and healthy donors	(van der Meer et al., 2017)

• NETs were observed as early as 30 min post-infection and contained active MPO and ROS.<br>• NETs size increased in time and reached a maximum level of expression at 6–8h after infection.<br>• All life *M.tb*, inactivated *M.tb*, lipid components from *M.tb*, *M. bovis* BCG and *M. smegmatis* were shown to induce the formation of NETs.	NET	*M.tb*	*In vivo* and *invitro* (blood and skin samples fromguinea pigs)	• ROS	Early stage of infection (0-6h)	(Filio-Rodríguez et al., 2017)
• *M.tb* induces NETosis.<br>• Mycobacterial antigens ESAT-6 and CFP-10 induced NETosis.	NET	*M.tb*	*In vivo* (sera from ATB patients)	• ROS<br>• Phagocytosis	ATB	(Rojas-Espinosa et al., 2021)
• Rv0888 sphingomyelinase activity was found to induce the formation of NETs *in vitro*and *in vivo*. Those NET structures observed in the lung tissue lacked mesh-like extracellular chromatin structures, possibly due to the space limitations of the lung parenchyma.<br>• Increased levels of IL-6, TNF-α, and IL-1β were associated with NETs-mediated lung injury.	NET	Recombinant *M. smegmatis* Rv0888	*In vivo* (C57BL/6 mice)	• Apoptosis<br>• Phagocytosis	ATB	(Dang et al., 2018)
• Increased levels of plasma MPO-DNA, MPO, and NE were significantly higher in patients with ATB compared to subjects with LTBI and healthy controls, and correlated with mycobacterial burden.	NET	*M.tb*	*In vivo* (human TB patients)	-	ATB, LTBI	(Schechter et al., 2017)
• *M.tb* induces NET formation which does not adhere to the shape of the neutrophil nuclei.<br>• MMP-8 and MMP-9 are up-regulated in TB patients and caused matrix destruction. Similar levels of MMP-8 were found after stimulation with CoMTB-stimulated neutrophils.	NET	*M.tb*	*In vitro* (neutrophils infected with *M.tb in vitro*) and *in vivo* (respiratory samples)	-	ATB	(Ong et al., 2015)

• Elevated levels of MMP-8 in TB sputum samples.<br>• α1AT is an important negative modulator of the NET activity<br>• NET formation was the main neutrophil's mechanism of action responsible for LTD due to TB infection in the studied cohort.<br>• Patients with higher cit-H3 levels exhibited increased cavity formation.<br>• Cit-H3 is a potent marker for NET formation and therefore probably a marker for LTD in TB.	NET	*M.tb*	*In vivo* (serum from patients with pulmonary TB)	• ROS<br>• Phagocytosis	ATB	(de Melo et al., 2019)
• NETs induced by BCG contained active LL-37<br>• NET formation upon stimulation with BCG is ROS-dependent<br>• NET cathelicidin is internalized by human macrophages through active endocytosis and is then transported into macrophage lysosomes.	NET	BCG	*In vitro* (human monocyte-derived macrophages and neutrophils)	• ROS<br>• Phagocytosis	Healthy blood donors	(Stephan et al., 2016)
• ALF-exposed *M.tb* has limited ability to induce NET formation.	NET	*M.tb*	Human alveolar lining fluid and neutrophils	-	Healthy blood donors	(Arcos et al., 2015)
METs by *M.tb*-stimulated macrophages structure were similar to those resulting from activated neutrophils and its formation was regulated by elastase activities.<br>• MET formation was more efficient by mycobacterial clumps.<br>• IFN-γ was found to enhance *M.tb*–induced METs which depends on ESX-1.	MET	*M.tb* (H37Rv and ΔESX-1)	*In vitro* (THP-1 macrophages) and *in vivo* (human macrophages from healthy donors)	• Cell death, elastase involved	Healthy blood donors	(Wong and Jacobs, 2013)
• Macrophages infected with non-cording bacteria produced relatively less METs compared to those released from cording-*M.tb.*<br>• METs by cording-*M.tb* were formed either in the form of threads or meshwork<br>• MET formation was shown not to be ROS-dependent in hMDMs.<br>• ESAT-6 is essential for MET formation in *M.tb*-infected macrophages.	MET	*M.tb* (H37Rv and (H37Rv -ΔESAT -6)	*In vitro* (hMDMs)	• Independent of ROS	Healthy blood donors	(Kalsum et al., 2017)

hN, human neutrophils; LDG, low density granulocytes; NDG, normal density granulocytes; ALF, human alveolar lining fluid; *M.tb*, *mycobacterium tuberculosis*; TB, tuberculosis; BCG, bacillus Calmette-Guérin; LTBI, latent

tuberculosis infection; ATB, active tuberculosis;; ETs, extracellular traps; ROS, reactive oxygen species; NETs, neutrophil extracellular traps; METs, macrophage extracellular traps; ESX-1, ESAT-6 secretion system-1; NE, neutrophil elastase; PMA, phorbol 12-myristate 13-acetate; hMDMs, human monocyte-derived macrophages; GM-CSF, granulocyte-macrophage colony-stimulating factor; CoMTB, conditioned media from *M.tb*-infected monocytes; α1AT, alpha-1-antitrypsin; LTD, lung tissue damage
